# The relationship between national identity and public health support needs to be reconsidered

**DOI:** 10.3389/fpsyg.2025.1454434

**Published:** 2025-03-07

**Authors:** Junhua Dang, Hongfei Du, Zhihao Ma, Lili Xu, Tuo Liu, Xiangyang Bi

**Affiliations:** ^1^Faculty of Humanities and Social Sciences, Xi'an Jiaotong University, Xi'an, China; ^2^Department of Surgical Sciences, Uppsala University, Uppsala, Sweden; ^3^Department of Psychology, Faculty of Arts and Sciences, Beijing Normal University at Zhuhai, Zhuhai, China; ^4^Beijing Key Laboratory of Applied Experimental Psychology, National Demonstration Center for Experimental Psychology Education (Beijing Normal University), Faculty of Psychology, Beijing Normal University, Beijing, China; ^5^Center for Studies of Psychological Application, South China Normal University, Guangzhou, China; ^6^Computational Communication Collaboratory, School of Journalism and Communication, Nanjing University, Nanjing, China; ^7^School of Psychology, Key Laboratory for Applied Statistics of MOE, Northeast Normal University, Changchun, China; ^8^Institute of Psychology, Goethe University Frankfurt, Frankfurt am Main, Germany; ^9^School of Ethnology and Sociology, Minzu University of China, Beijing, China

**Keywords:** national identity, public health, finite mixture modeling, physical hygiene, spatial distancing

## Introduction

The relationship between national identity and public health compliance has emerged as a critical topic in behavioral science, particularly during global crises like the COVID-19 pandemic. National identity, broadly defined as an individual's psychological attachment to their nation (Smith, 1991), is theorized to foster prosocial behaviors by reinforcing collective norms and shared responsibility (Brewer, 2001). Van Bavel et al. (2022) advanced this perspective by demonstrating that national identity robustly predicted compliance with public health measures across 67 countries. Based on personal level data collected from a large-scale international self-report survey (*N* = 49,968), Study 1 found national identity strongly predicted more spatial distancing, physical hygiene, and support for public health policies. Study 2 utilized aggregrated data at the national level (*N* = 42) and showed that national identity was associated with reduced mobility trends to outside places. The authors thus claimed that understanding the role of national identity was essential to address public health crises.

While their work underscores the potential of national identity as a unifying force, it leaves unresolved questions about the universality of this relationship. For instance, cultural psychology research highlights that national identity operates differently across societies: in “honor-shame” cultures, compliance may stem from external social pressures, whereas in “dignity-guilt” cultures, internalized moral values might dominate (Leung and Cohen, 2011). Such distinctions suggest that Van Bavel et al.'s global analysis risks oversimplifying culturally contingent mechanisms.

Moreover, methodological limitations inherent to self-reported compliance data warrant scrutiny. Social desirability bias—wherein respondents overreport adherence to socially approved behaviors—has been extensively documented in health surveys (Krumpal, 2013). During the pandemic, this bias likely intensified due to moralized public narratives framing noncompliance as a civic failure (Kunnari et al., 2024). For example, Mieth et al. (2021) found significant discrepancies between self-reported hygiene practices and objective sensor data, implying that survey measures may conflate genuine compliance with performative responses. These issues raise doubts about whether observed correlations reflect true behavioral patterns or methodological artifacts.

Considering the significance of Van Bavel et al.'s (2022) work in social and behavioral sciences, we reexamined this topic by reanalyzing their data and found that the results were not as robust as claimed by Van Bavel et al. By employing the finite mixture modeling (FMM) to investigate potential subgroups of the three key outcome indicators of Study 1, we observed much weaker relationships between national identity and these outcome indicators. Meanwhile, when an important covariate (i.e., human development index, HDI), which was also considered by Van Bavel et al. in Study 1, was controlled in their data of Study 2, the correlation between national identity and mobility at the national level fell short of significance. Overall, the results from our re-analyses suggest the predictive effect of national identity on pubic health support may have been greatly overestimated.

## Analysis

**Figure 1:**
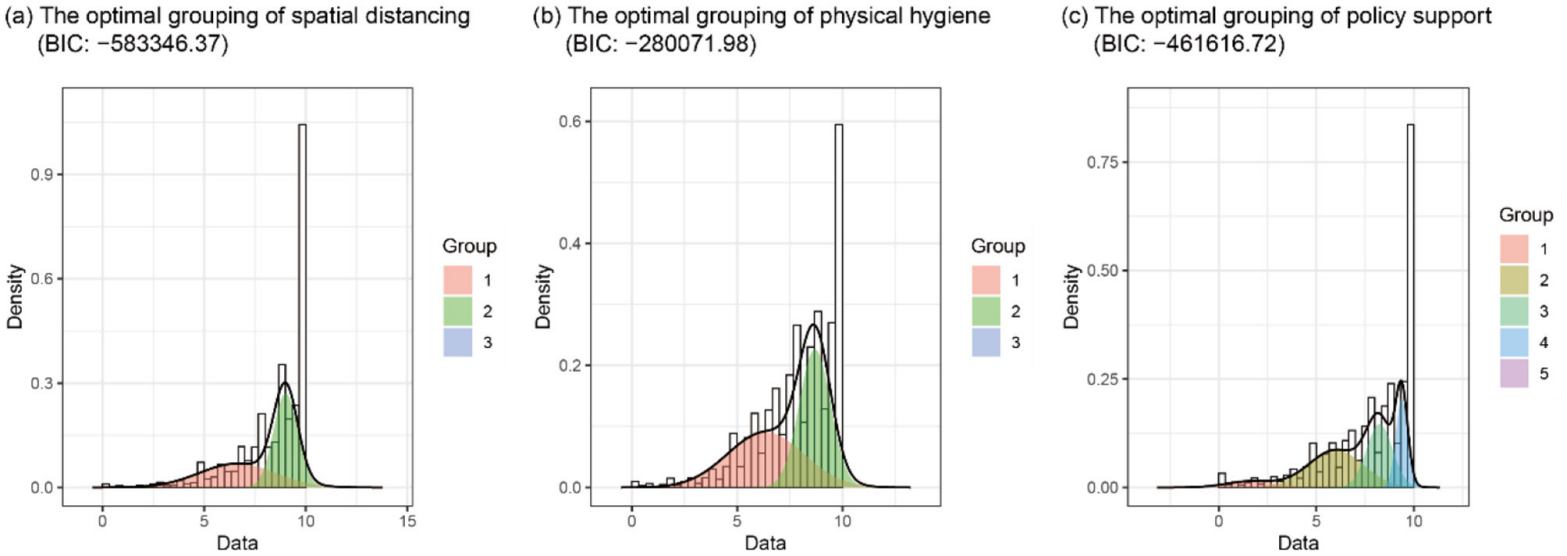
The optimal number of finite mixture models on three outcome variables. Group 3 in **(A, B)** and group 5 in **(C)** are not shown because they are made up of the participants who reported the highest score.

In Van Bavel et al.'s Study 1, all the three outcome indicators of public health support (i.e., spatial distancing, physical hygiene, and policy support) suffered from extreme skewed distributions, with all means above 8 on a scale ranging from 0 to 10 (Figure 1). Although the extreme skewed data may reflect that “many people supported initiatives to mitigate the spread of COVID-19” (J. Van Bavel, personal communication, April 6, 2023), we argue that it can also indicate that the measures of the outcome indicators may have been greatly contaminated by various biases, thus resulting in distorted estimations of the relationships between national identity and the outcome indicators. On one hand, the contamination may have resulted from social desirability. During such a special context as the COVID-19 pandemic, it was very likely that participants rated the question in a way that might indicate their desire to be seen as good citizens or to adhere to “political correctness,” rather than reflect their true thoughts and behaviors (Daoust et al., 2021; Mieth et al., 2021). On the other hand, participants' ratings may also have been influenced by many response biases, such as the acquiescence bias (the tendency to agree with survey items regardless of the question's content) and the extreme bias (the tendency to select end-points of the response scale) (Baron-Epel et al., 2010).

Although it is difficult to explicitly distinguish participants who responded according to their true thoughts and behaviors from those whose responses may have been biased, there are mathematical tools that can categorize participants into different subgroups defined by unobservable variables. That is, while a group of people can often be divided into subgroups based on observable variables such as age (i.e., old vs. young) and sex (man vs. woman), sometimes a variable that can identify potential subgroups is difficult to collect or is inherently unobservable. In such cases, the finite mixture modeling (FMM) can be used to model participants' probability of belonging to each unobserved subgroup (McLachlan et al., 2019).

We used the R package *mixR* to conduct the FMM and investigated potential unobserved heterogeneous subgroups for all the three outcome indicators (Yu, 2022). The optimal number of latent subgroups was selected based on the Bayesian Information Criterion (BIC). If the predictive effect of national identity was robust, it should be manifested in each of the subgroups and comparable to the result derived from the whole sample. As shown in Figure 1, the optimal number of latent subgroups for spatial distancing and physical hygiene was 3, while the optimal number of latent subgroups for policy support was 5. For all the three outcome indicators, a subgroup scoring “10” on the scale emerged, which could not be analyzed because the outcome in this subgroup was a constant. We then repeated Van Bavel et al.'s analyses (i.e., multilevel regressions with each of the three outcome indicators as the dependent measure; natonal identity, national narcissism and political ideology as predictors and randomly varying; and country as the cluster variable) in the remaining subgroups.

As shown in Table 1, national identity was positively associated with the outcome variables in only 5 out of 8 subgroups that can be analyzed (there are still three subgroups scoring “10” that cannot be analyzed). Meanwhile, the coefficients shank greatly, ranging from 0.004 to 0.086, compared to a range from 0.126 to 0.129 in Van Bavel et al.'s results. The marginal *R*^2^s, which reflect the proportions of variance explained by the three predictors as fixed factors, were very small (ranging from 0.001 to 0.017). We followed the procedure of Van Bavel et al. and controlled for the human development index (HDI), which reflects a country's human development in three key dimensions: health, knowledge and standard of living. The analyses of including HDI as a covariate yielded the same results.

**Table 1:** Multilevel regression results for subgroups derived from the FFM.

	**Spatial distancing**		**Physical hygiene**		**Policy support**			
	**Group 1**	**Group 2**	**Group 1**	**Group 2**	**Group 1**	**Group 2**	**Group 3**	**Group 4**
Intercept	**6.165**	**9.025**	**5.817**	**8.757**	**1.301**	**5.714**	**8.151**	**9.370**
National narcissism	−0.004	−0.006	0.031	**0.015**	0.007	0.015	0.002	0.000
National identity	**0.086**	**0.022**	**0.061**	**0.020**	0.005	**0.030**	0.007	0.004
Political ideology	−0.003	**–0.010**	−0.015	−0.004	0.004	−0.008	−0.004	−0.001
*MarginalR* ^2^	0.017	0.008	0.017	0.012	0.001	0.006	0.002	0.001
Conditional *R*^2^	0.111	0.053	0.075	0.067	0.074	0.040	0.022	0.032
ICC	0.067	0.034	0.034	0.049	0.049	0.021	0.018	0.028
Num. obs.	12,666	21,696	16,107	23,326	1,937	13,193	11,145	9,260

Bold numbers indicate coefficients that were significant at *p* < 0.001, which was set by the original authors.

To theoretically replicate the findings in Study 1, Van Bavel et al.'s Study 2 reported a strong correlation between a national identity index derived from the World Value Survey and an index of mobility trends to outside places derived from the Google Community Mobility Reports at the national level (*r* = −0.40, *p* = 0.008). It is worthwhile to note that Van Bavel et al. controlled for HDI in Study 1 but not in Study 2. We argue that as a theoretical replication of Study 1, Study 2 should also rule out the potential confounding impact of HDI on public health support. Therefore, it is essential to include HDI as a covariate in not only Study 1 but also Study 2 to examine the robustness of the results. Surprisingly, when we used partial correlation to control for HDI, the correlation between national identity and mobility became insignificant (partial *r* = −0.29, *p* = 0.063). Therefore, the results of Study 2 of Van Bavel et al. fell short of significance when we included the same covariate in Study 1 (i.e., HDI).

## Discussion

Our reanalyses challenge the robustness of Van Bavel et al.'s (2022) conclusions, revealing that the relationship between national identity and public health support diminishes substantially when accounting for latent subgroups and socioeconomic confounders like HDI. These findings align with critiques of universalist models in behavioral science, which often neglect cultural and economic heterogeneity (Muthukrishna and Henrich, 2019). For instance, the weakened associations in FMM-derived subgroups suggest that national identity may primarily motivate compliance among individuals with moderate adherence levels, while having negligible effects on polarized subgroups (e.g., maximal compliers or non-compliers). This mirrors Gelfand et al.'s (2021) observation that “tight” cultures with strong norms exhibit higher compliance, whereas “loose” cultures require tailored interventions. In addition, the insignificance of national identity in HDI-adjusted analyses further underscores the primacy of structural factors. Countries with higher HDI scores typically exhibit greater institutional trust and resource accessibility (Inglehart and Welzel, 2005), which may overshadow identity-driven motivations.

Methodologically, our use of FMM demonstrates its utility in addressing survey biases—a lesson with broad implications for public health research. Future studies could integrate FMM with longitudinal designs to track subgroup dynamics across pandemic phases or combine it with qualitative methods to explore psychosocial drivers of compliance (e.g., interviews differentiating performative versus genuine adherence). Limitations of our study include reliance on secondary data, which precluded testing additional moderators like political trust or cultural tightness. Nevertheless, these findings urge policymakers to adopt adaptive strategies aligned with the World Health Organization (2023) emphasis on equity-centered communication.
